# Transcriptome analysis reveals that exogenous ethylene activates immune and defense responses in a high late blight resistant potato genotype

**DOI:** 10.1038/s41598-020-78027-5

**Published:** 2020-12-04

**Authors:** Xiaohui Yang, Li Chen, Yu Yang, Xiao Guo, Guangxia Chen, Xingyao Xiong, Daofeng Dong, Guangcun Li

**Affiliations:** 1 Institute of Vegetables and Flowers, Shandong Academy of Agricultural Sciences, Molecular Biology Key Laboratory of Shandong Facility Vegetable, National Vegetable Improvement Center Shandong Sub-Center, Huang-Huai-Hai Region Scientific Observation and Experimental Station of Vegetables, Ministry of Agriculture and Rural Affairs, Jinan, 250100 China; 2 Institute of Vegetables and Flowers, Chinese Academy of Agricultural Sciences, Key Laboratory of Biology and Genetic Improvement of Tuber and Root Crop, Ministry of Agriculture and Rural Affairs, Beijing, 100081 China

**Keywords:** Plant sciences, RNA sequencing, Gene expression

## Abstract

Ethylene (ET) is one of the many important signaling hormones that functions in regulating defense responses in plants. Gene expression profiling was conducted under exogenous ET application in the high late blight resistant potato genotype SD20 and the specific transcriptional responses to exogenous ET in SD20 were revealed. Analysis of differentially expressed genes (DEGs) generated a total of 1226 ET-specific DEGs, among which transcription factors, kinases, defense enzymes and disease resistance-related genes were significantly differentially expressed. GO enrichment and KEGG metabolic pathway analysis also revealed that numerous defense regulation-related genes and defense pathways were significantly enriched. These results were consistent with the interaction of SD20 and *Phytophthora infestans* in our previous study, indicating that exogenous ET stimulated the defense response and initiated a similar defense pathway compared to pathogen infection in SD20. Moreover, multiple signaling pathways including ET, salicylic acid, jasmonic acid, abscisic acid, auxin, cytokinin and gibberellin were involved in the response to exogenous ET, which indicates that many plant hormones work together to form a complex network to resist external stimuli in SD20. ET-induced gene expression profiling provides insights into the ET signaling transduction pathway and its potential mechanisms in disease defense systems in potato.

## Introduction

Plant hormones are important signal molecules that function in regulating biotic and abiotic stress responses^1,2^. Ethylene (ET) is a stress–responsive hormone in addition to regulating plant growth and development. Infestation with *Magnaporthe oryzae* activates biosynthesis of ET in rice, and the ET content accumulated in the resistant rice varieties is higher than that in susceptible rice varieties^3^. *Cochliobolus miyabeanus* inhibits host defense by hijacking the ET signaling pathway, which interferes with the synthesis and/or activation of ET, and significantly enhances rice resistance to brown spot disease^4^. After infection by the super race isolate CN152 of *Phytophthora infestans (Pi*) in the tetraploid potato genotype SD20, multi-signaling pathways of salicylic acid (SA), jasmonic acid (JA), and ET were involved in resistance and defense against *Pi*, and one ET synthesis pathway enzyme, 1-aminocyclopropane-1-carboxylate oxidase (ACO), was enhanced in *Pi* infected potato^5^. In addition, the pathogenesis-related (PR) protein 5 Osmotin gene *OSML15* (PGSC0003DMG400003057) was down-regulated in *Pi* 90128- and CN152-infected potato samples^6^.

Exogenous application of plant hormones can improve plant resistance. ET-induced transgenic rice shows broad-spectrum resistance to fungal pathogens *M. oryzae* and *Rhizoctonia solani*^7^. The signaling pathway regulated by ET can induce the expression of defensive genes to enable plants to produce local or systemic defenses^8,9^. The defensive gene *StOsmotin2* was specifically induced by ET in potato cultivar Desiree^9^. ET signaling regulates the relative abundance of the two *Fusarium graminearum*-resistance-related metabolites smilaside A (3,6-diferuloyl-3′,6′-diacetylsucrose) and smiglaside C (3,6-diferuloyl-2′,3′,6′-triacetylsucrose) and affects resistance against *F. graminearum* in maize seedling roots^10^.

Potato (*Solanum tuberosum*) is the third largest food crop in the world after wheat and rice. Potato production is affected by a variety of abiotic and biotic stresses. Most of the knowledge of hormone biology in plants is based on work done in *Arabidopsis* and rice, while only a few studies have been performed to show the effects of different hormones at the whole genome level in potato^5,11^. Wiesel et al.^9^ generated a transcriptional reference map of the early potato responses to exogenous application of abscisic acid (ABA), brassinolides, ET, SA and JA, and identified some specific marker genes for early hormone responses in *S. tuberosum* cultivar Desiree using microarray analysis. In a previous study, we obtained the tetraploid potato genotype SD20 showing high resistance to *P. infestans* super race CN152, and systematically analyzed the gene expression induced by the pathogen. Multiple signaling pathways of ET, JA, and SA were involved in the immune defense response to CN152^5^. To further investigate specific transcriptional responses to exogenous ET in the genotype SD20, SD20 was treated with exogenous ET (supplied with 0.2 mmol·L^−^^1^ ethephon solution) at different processing times followed by RNA-seq analysis. The goal of this study was to explore the expression of ET-induced early response genes and their biological processes in the defense response, to provide a reference in the application of plant hormones in the potato defense response.

## Results

### RNA-sequencing and mapping

In order to obtain the spectrum of gene expression of SD20 in the response to ET treatment, transcription profiling was conducted in SD20 treated with exogenous ET for 0, 3, 6, and 12 h and compared with H_2_O as the mock treatment. Twenty-one RNA libraries derived from seedling samples of 3, 6, and 12 h including mock (0 h H_2_O) with three biological replicates were sequenced using the Illumina HiSeq X Ten system with the 150-cycle paired-end sequencing protocol. After data filtering and quality assessment, approximately 142.43 Gb clean data were produced, yielding a range of 6.01–8.52 Gb clean bases per sample. The Q30 percentage (sequencing error rate < 0.1%) was above 91.14%. When mapping the RNA-seq reads to the potato doubled haploid DM reference genome, 83.48–89.15% of the reads could be mapped and 79.97–86.19% could be mapped uniquely to one location (Table 1).

**Table 1:** Basic summary of RNA-sequencing results.

Samples	Total reads	Total bases (Gb)	Mapped reads	Uniquely mapped reads	% ≥ Q30
H_2_O_0h_rep1	48,887,878	7.33	42,934,968 (87.82%)	41,505,712 (84.90%)	94.59%
H_2_O_0h_rep2	40,648,794	6.10	35,505,668 (87.35%)	34,372,398 (84.56%)	95.05%
H_2_O_0h_rep3	49,497,462	7.42	43,507,054 (87.90%)	42,046,007 (84.95%)	95.22%
H_2_O_3h_rep1	42,446,212	6.37	37,811,744 (89.08%)	36,520,430 (86.04%)	95.04%
H_2_O_3h_rep2	48,459,112	7.27	41,599,539 (85.84%)	40,422,493 (83.42%)	95.29%
H_2_O_3h_rep3	56,784,932	8.52	48,660,422 (85.69%)	47,157,977 (83.05%)	95.02%
H_2_O_6h_rep1	49,773,000	7.47	43,609,541 (87.62%)	42,367,182 (85.12%)	94.78%
H_2_O_6h_rep2	40,098,078	6.01	33,472,987 (83.48%)	32,067,312 (79.97%)	91.14%
H_2_O_6h_rep3	43,758,884	6.56	36,862,096 (84.24%)	35,806,520 (81.83%)	95.41%
H_2_O_12h_rep1	45,843,118	6.88	39,840,187 (86.91%)	38,632,684 (84.27%)	95.06%
H_2_O_12h_rep2	41,864,734	6.28	36,868,757 (88.07%)	35,522,910 (84.85%)	94.56%
H_2_O_12h_rep3	41,780,560	6.27	36,886,851 (88.29%)	35,496,813 (84.96%)	94.68%
ET_3h_rep1	46,906,526	7.04	41,674,506 (88.85%)	40,263,288 (85.84%)	95.13%
ET_3h_rep2	41,467,594	6.22	36,900,345 (88.99%)	35,703,274 (86.10%)	95.20%
ET_3h_rep3	40,874,040	6.13	35,997,450 (88.07%)	34,789,903 (85.11%)	95.15%
ET_6h_rep1	44,701,810	6.71	39,583,201 (88.55%)	38,217,813 (85.50%)	95.46%
ET_6h_rep2	47,274,804	7.09	42,143,691 (89.15%)	40,734,174 (86.16%)	95.21%
ET_6h_rep3	50,731,616	7.61	45,145,424 (88.99%)	43,668,253 (86.08%)	94.47%
ET_12h_rep1	41,454,374	6.22	36,242,254 (87.43%)	35,030,002 (84.50%)	94.89%
ET_12h_rep2	45,021,372	6.75	39,320,532 (87.34%)	37,935,710 (84.26%)	94.87%
ET_12h_rep3	41,280,524	6.19	36,742,730 (89.01%)	35,578,963 (86.19%)	94.73%

### Differentially expressed genes in the response to exogenous ET

**Figure 1:**
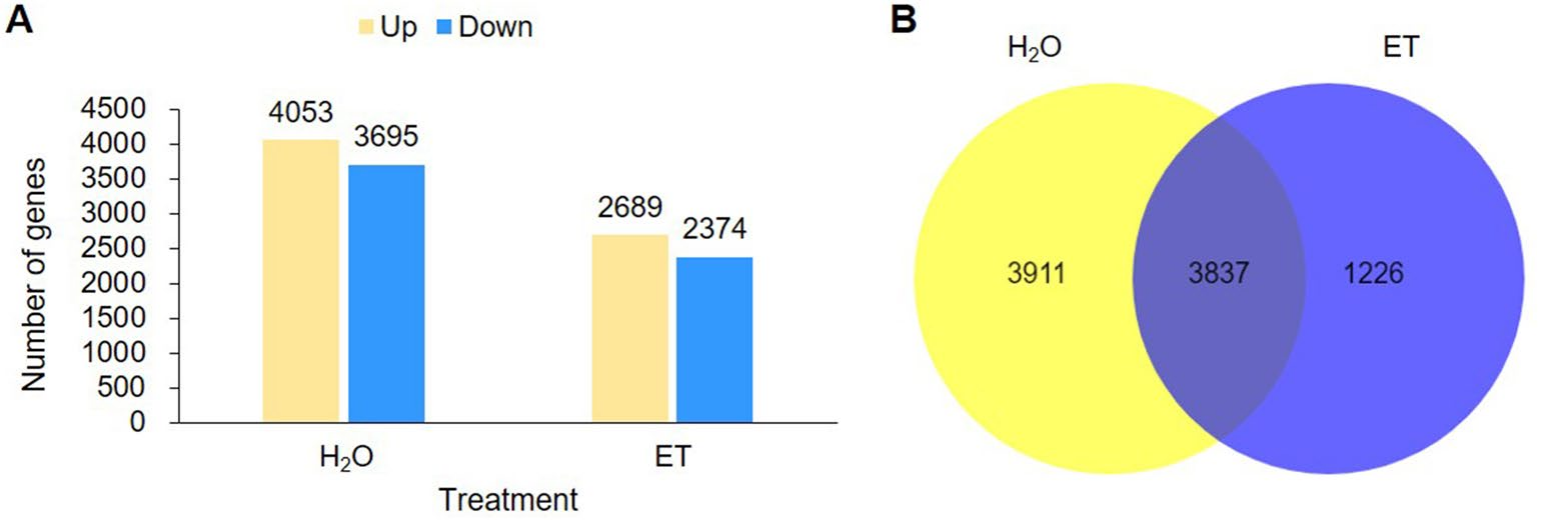
Summary of DEGs in potato genotype SD20 under H_2_O and ethylene (ET) treatments. (**A**) The summary of DEGs in a bar graph. (**B**) Venn diagram of DEGs identified in SD20.

The levels of gene expression were estimated by FPKM (Fragments per kilobase of transcript per million mapped reads). Differential expression analysis was performed using DESeq. Genes with a false discovery rate (*FDR*) < 0.05 and a |log_2_ (fold change) |≥ 1 were set as differentially expressed. The differentially expressed genes (DEGs) were detected by performing pairwise comparisons at each time point within a single treatment. In total, 7748 and 5063 DEGs were identified for the H_2_O and ET treatments, respectively (Fig. 1A). Further comparison of DEGs in the two treatments, 3911 genes were specifically differentially expressed between any two time points only in H_2_O treatment, and 1226 genes were specifically differentially expressed upon ET treatment (Fig. 1B), 754 of which were up-regulated and 472 of which were down-regulated (Table 2).

**Table 2:** Differentially expressed genes unique to ethylene treatment.

Treatment (h)	Total	Up	Down
0 vs 3	264	107	157
0 vs 6	595	347	248
0 vs 12	324	268	56
3 vs 6	9	7	2
3 vs 12	16	11	5
6 vs 12	18	14	4
Total	1226	754	472

### GO enrichment analysis revealed numerous defense regulation-related genes that were enriched in potato host response to ET

**Figure 2:**
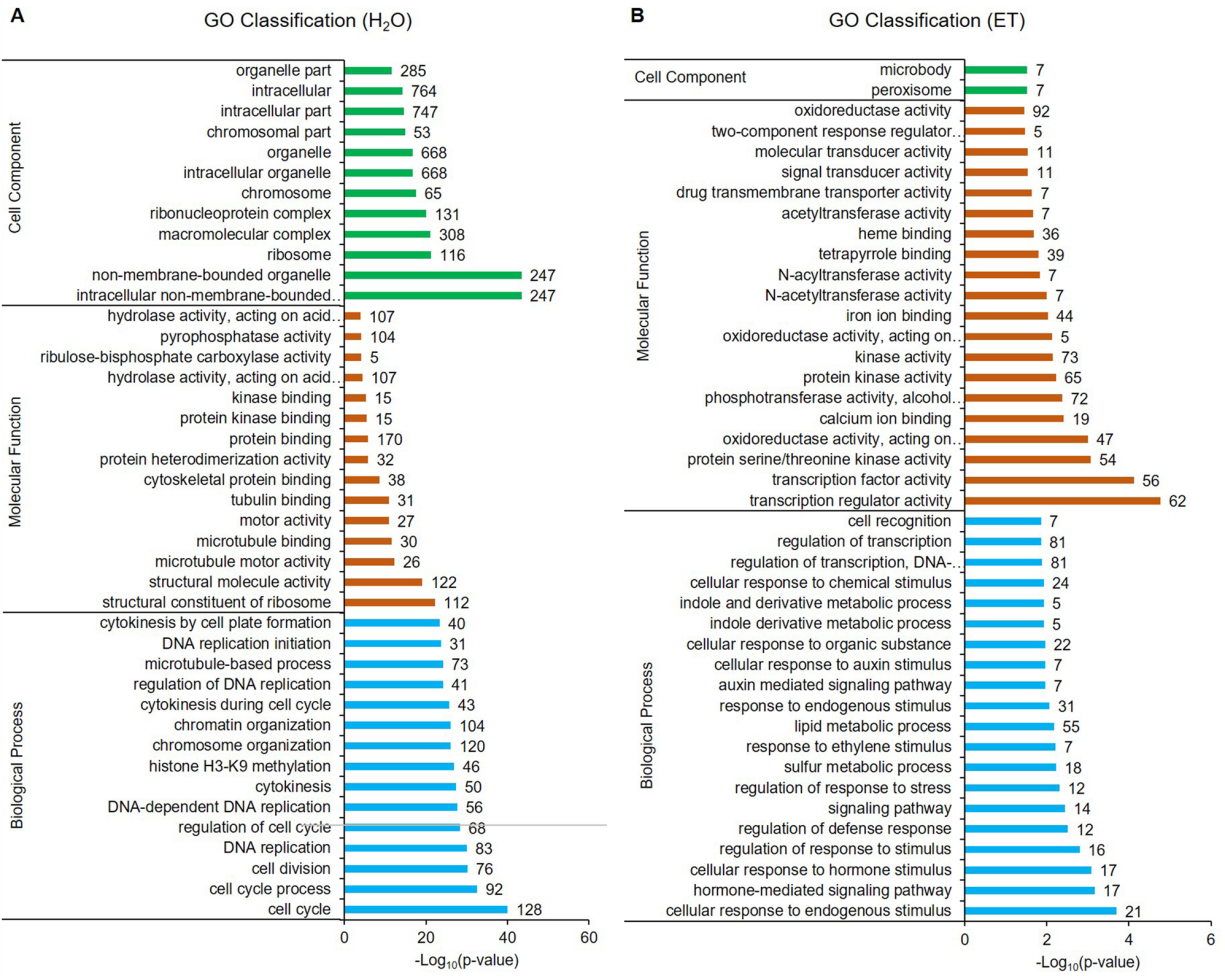
Gene ontology analysis of specifically differentially expressed genes under H_2_O (**A**) and ethylene (**B**) treatment.

DEGs that specifically responded to H_2_O and ET treatments were annotated using gene ontology (GO) analysis. A total of 498 and 92 GO terms were significantly enriched in different biological process, molecular function, and cell component categories in water and ethylene treatments, respectively (*p* value < 0.05). The top 10–20 enriched terms are in Fig. 2.

In the control, 3911 DEGs were categorized into 498 functional GO terms. In biological processes, 384 GO terms were significantly enriched. The top GO terms were cell cycle (GO:0007049, 128 DEGs), cell cycle process (GO:0022402, 92 DEGs), and cell division (GO:0051301, 76 DEGs) (Fig. 2A). The most DEGs were enriched in metabolic process (1237 DEGs), followed by cellular process (1217 DEGs) and cellular metabolic process (984 DEGs). In molecular function, a total of 32 GO terms were significantly enriched, including structural constituent of ribosome (GO:0003735, 112 DEGs), structural molecule activity (GO:0005198, 122 DEGs), and microtubule motor activity (GO:0003777, 26 DEGs) (Fig. 2A). The most DEGs were enriched in nucleic acid binding (382 DEGs), DNA binding (263 DEGs), and protein binding (170 DEGs).

**Figure 3:**
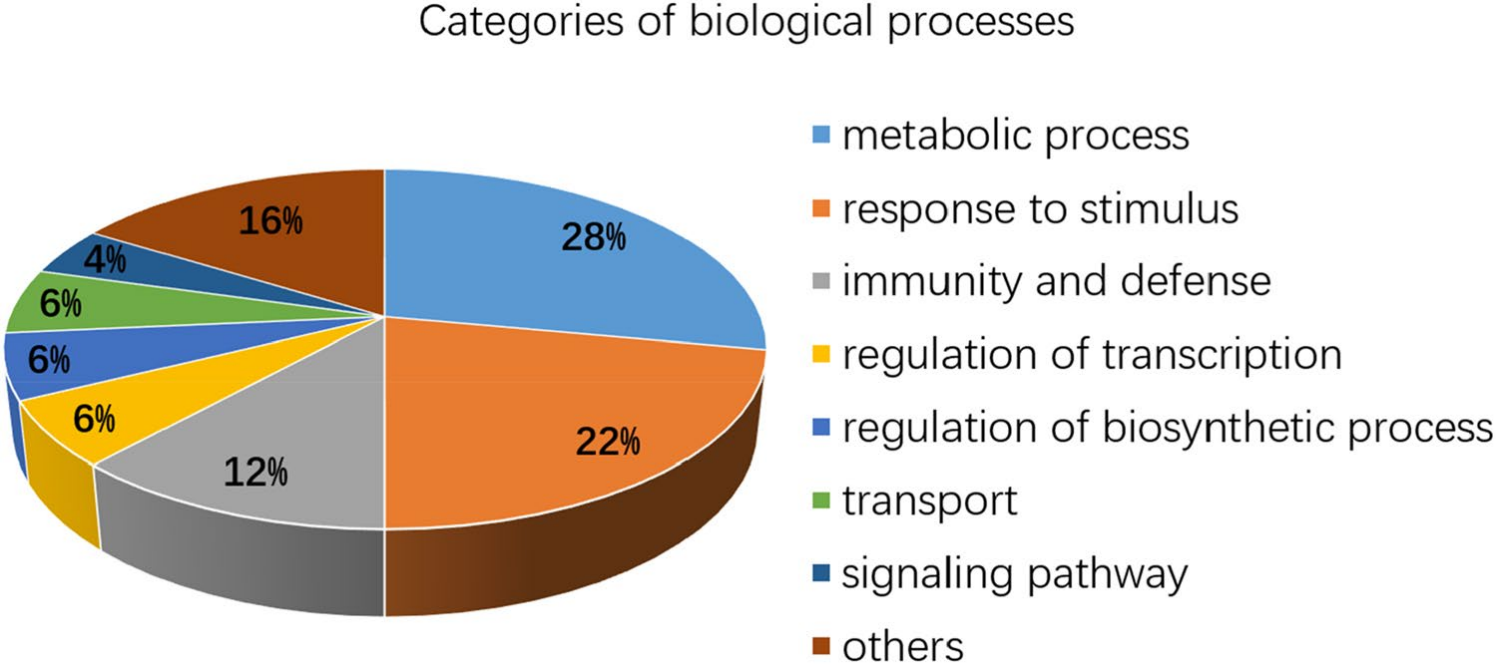
Categories of biological processes enriched in the differentially expressed genes under ethylene treatment.

In the ET treatment, the 1226 unique DEGs were specifically (*p *value < 0.05) annotated into 92 GO terms, including 68 biological process, 22 molecular function, and two cellular component categories. The top 20 GO terms in biological process and molecular function are shown in Fig. 2B. Sixty-eight biological processes were divided into categories including metabolic process, response to stimulus, immunity and defense, regulation of transcription, regulation of biosynthetic process, transport, and others (Fig. 3). Metabolic process was the largest category, containing 19 terms (28%), including cell wall macromolecule (GO:0044036, 12 members), lipid (GO:0006629, 55 members), indole derivative (GO:0042434, five members), and phosphate (GO:0006796, 81 members). The second category was GO terms with response to stimulus (15 terms, 22%), including cellular response to endogenous stimulus (GO:0071495), cellular response to hormone stimulus (GO:0032870), response to auxin stimulus (GO:0009733), and response to ET stimulus (GO:0009723). The third category was related to immunity and defense (eight terms, 12%), including regulation of immune system process (GO:0002682, eight DEGs), regulation of innate immune response (GO:0045088, eight DEGs), regulation of defense response (GO:0031347, 12 DEGs), regulation of plant-type hypersensitive response (GO:0010363, six DEGs), host programmed cell death induced by symbiont (GO:0034050, seven), plant-type hypersensitive response (GO:0009626, seven DEGs), regulation of response to stress (GO:0080134, 12 DEGs), and regulation of immune response (GO:0050776, eight DEGs).

In the molecular function category, the top GO terms were transcription regulator activity (GO:0030528, 62 members), transcription factor activity (GO:0003700, 56 members), protein serine/threonine kinase activity (GO:0004674, 54 members), oxidoreductase activity (GO:0016705, 47 members), and calcium ion binding (GO:0005509, 19 members) (Fig. 2B). In the cellular component category, DEGs were enriched in terms of peroxisome (GO:0005777, seven members) and microbody (GO:0042579, seven members) (Fig. 2B), which indicated the enriched genes were mainly located on the oxidases and microbodies.

As above, specific DEGs in H_2_O treatment were mainly plant growth and development-related genes, and genes related to disease-resistance and regulation were not identified. ET treatment first initiated the response to stimulus, then mobilized the biological synthesis of various biological macromolecules, and finally regulated the expression of related genes through ion transport and signal transduction, which induced the immune defense process and may be related to disease resistance.

### KEGG metabolic pathway analysis reveals DEGs involved in multi-hormone signaling pathways and defense pathways in the response to ET

To better understand the interactions among DEGs in the potato SD20 response to ET, DEGs were compared to the KEGG database. The 1226 unique DEGs were annotated to 101 KEGG metabolic pathways, ten of which achieved significant differences with *p* value < 0.05 (Table 3), including plant hormone signal transduction (sot04075, 27 DEGs), plant-pathogen interaction (sot04626, 18 DEGs), and glutathione metabolism (sot00480, ten DEGs) (Table 3). The biosynthesis of secondary metabolites pathway had the most DEGs at 55. Nine DEGs were involved in the cysteine and methionine metabolism pathway, which was associated with the biosynthesis of ET substances.

**Table 3:** The KEGG pathways for 1226 differentially expressed genes under ET treatment.

KEGG Pathway	ID	Gene number	*p *value	Corrected *P-value*
Plant hormone signal transduction	sot04075	27	3.06E-05	0.0031
Plant-pathogen interaction	sot04626	18	0.0006	0.0303
Glutathione metabolism	sot00480	10	0.0058	0.1968
Nitrogen metabolism	sot00910	5	0.0116	0.2829
Sulfur metabolism	sot00920	5	0.0140	0.2829
Cysteine and methionine metabolism	sot00270	9	0.0209	0.3523
Biosynthesis of secondary metabolites	sot01110	55	0.0385	0.4680
Sesquiterpenoid and triterpenoid biosynthesis	sot00909	3	0.0444	0.4680
Phosphatidylinositol signaling system	sot04070	6	0.0451	0.4680
Glycosphingolipid biosynthesis – globo series	sot00603	2	0.0463	0.4680

**Figure 4:**
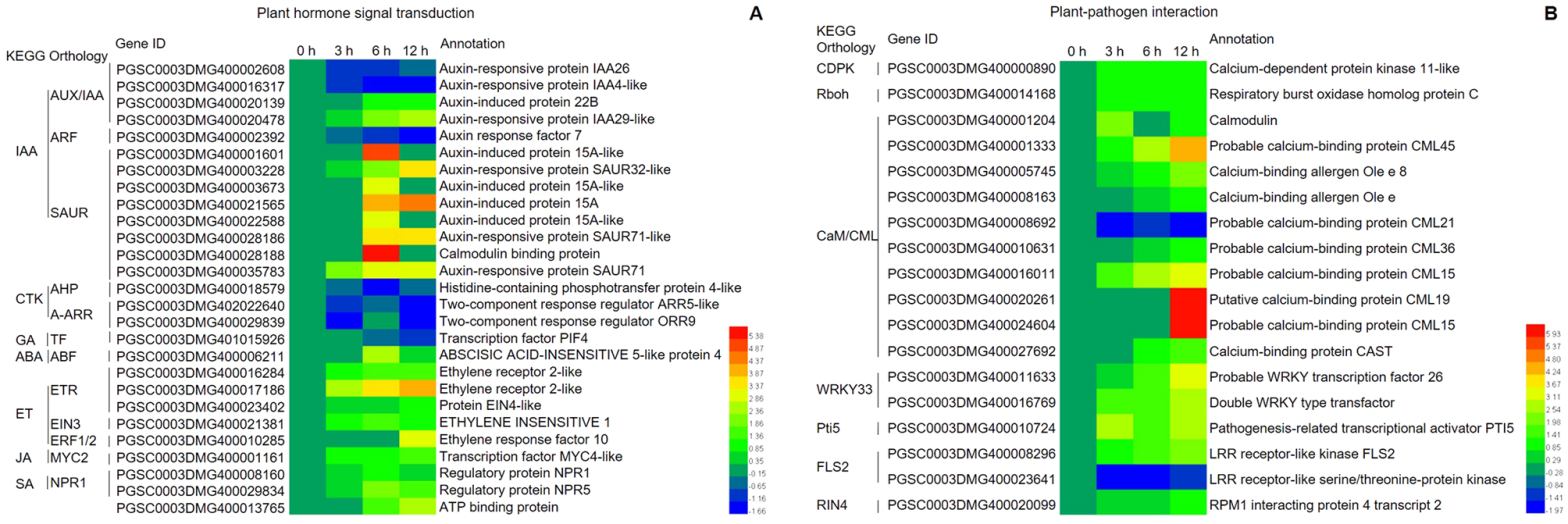
The expression trends and annotations of differentially expressed genes enriched in plant hormone signal transduction (**A**) and plant–pathogen interaction (**B**) pathways.

In the pathway of plant hormone signal transduction (sot04075)^12^, 27 DEGs encoded 12 functional proteins including auxin-responsive protein IAA (Aux/IAA), auxin response factor, histidine-containing phosphotransfer protein (AHP) and so on, which were involved in auxin (IAA), cytokinin (CTK), gibberellin (GA), ABA, ET, JA, and SA signal transduction pathways (Fig. 4A). Five DEGs were enriched in the ET signal transduction pathway, which encoded ethylene receptor ETR (K14509), ethylene-insensitive protein EIN3 (K14514), and ethylene-responsive transcription factor ERF1 (K14516) and were up-regulated. Two DEGs encoding regulatory protein NPR1 were enriched in the SA signal transduction pathway and also up-regulated. The genes that encoded phytochrome-interacting factor 4, ABA responsive element binding factor (AREB), and transcription factor MYC2 were enriched in the GA, ABA, and JA signal transduction pathways, respectively (Fig. 4A). The above results indicated that exogenous ET induced multi-hormone signal transduction pathways in SD20.

There were 18 DEGs in the plant-pathogen interaction pathway^12^ (Fig. 4B), of which ten genes encoded calmodulin/calcium-binding protein (CaM/CML), and the other eight genes encoded calcium-dependent protein kinase (CDPK), respiratory burst oxidase homologue (Rboh, also NADPH), WRKY33, pathogenesis-related genes transcriptional activator PTI5, LRR receptor-like serine/threonine-protein kinase FLS2, and RPM1-interacting protein 4 (RIN4). Among these 18 genes, only one CaM/CML coding gene (PGSC0003DMG400008692) and one FLS2 gene (PGSC0003DMG400023641) were down-regulated, the other 16 genes were up-regulated. This indicated that ET induced defense pathways in SD20.

A total of ten DEGs were enriched in the glutathione metabolism pathway (sot00480) and up-regulated. Among the ten DEGs, seven genes encoded glutathione S-transferase (GST), which is the key enzyme of the glutathione metabolism pathway, two genes encoded 6-phosphogluconate dehydrogenase (6-PGDH), and one gene encoded isocitrate dehydrogenase [NADP] (IDH), which indicated the important role of the glutathione metabolism pathway in response to ethylene stimulus in SD20.

### Exogenous ET induced the differential expression of genes in the endogenous ET synthesis pathway in SD20

**Figure 5:**
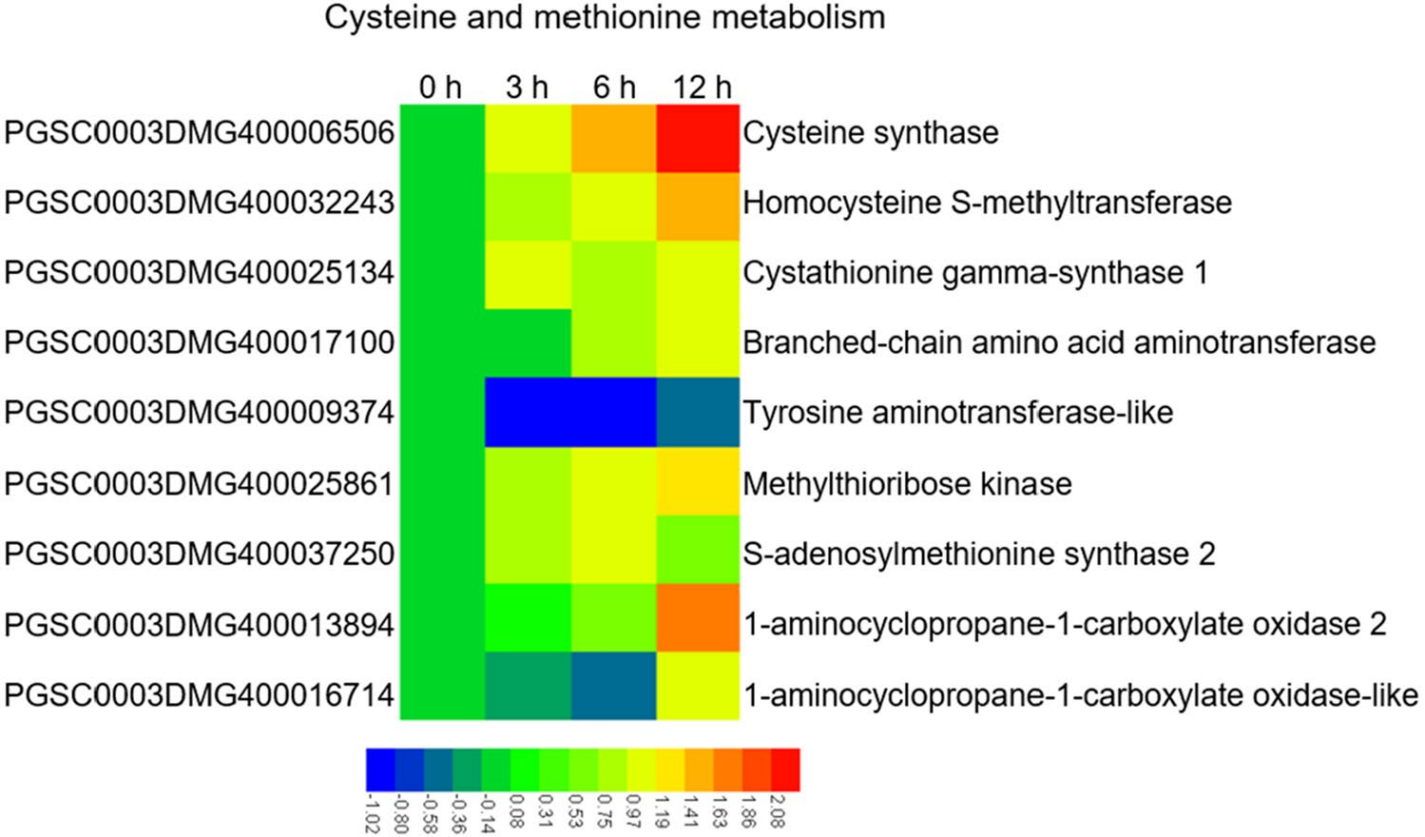
Gene expression and annotation of differentially expressed genes enriched in cysteine and methionine metabolism in potato SD20.

Cysteine and methionine metabolism (sot00270) contained nine DEGs, of which three genes encoded proteins closely related to ET biosynthesis: one S-adenosylmethionine synthase 2 (METK2) gene (PGSC0003DMG400037250) and two amino-cyclopropanecarboxylic acid (ACC) oxidase genes (PGSC0003DMG400013894 and PGSC0003DMG400016714), which were all up-regulated (Fig. 5). In addition, in the circulating metabolism of methionine, the ET precursor substance, the homocysteine S-methyltransferase gene (PGSC0003DMG400032243) and methylthioribose kinase (MTRK) gene (PGSC0003DMG400025861) were up-regulated, and the tyrosine aminotransferase-like gene (PGSC0003DMG400009374) was down-regulated (Fig. 5). This indicated that exogenous ET induced differential expression of key genes of the endogenous ET synthesis pathway in SD20.

### Differential expression of transcription factors response to ET

**Figure 6:**
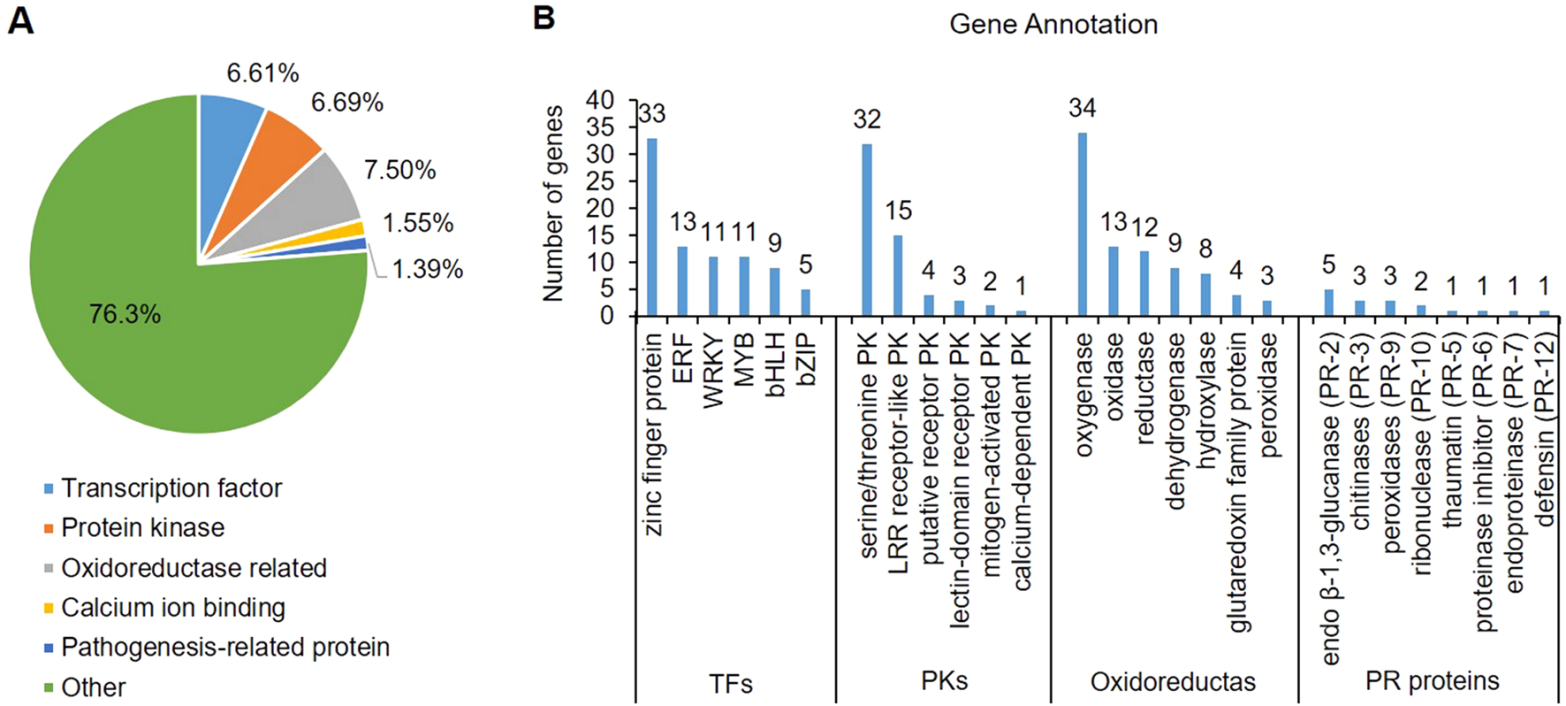
Differential expression of transcription factors, protein kinases, oxidoreductases and pathogenesis-related proteins that specifically respond to ET. (**A**) Category of differentially expressed genes. (**B**) The gene families for each category.

Out of the 1226 ET-specific DEGs, functional annotation showed that 81 genes encoded transcription factors (TFs), which belonged to 13 TF families (Fig. 6A). The largest group was zinc finger protein TFs with 33 members, followed by ET response factor (ERF, 13 members), WRKY (11 members), MYB (11 members), bHLH (nine members), bZIP (five members), and so on (Fig. 6B). Up to 72.8% of 81 TF genes were up-regulated with prolonged ET treatment time, indicating that most TF genes played a positive regulatory role in responding to exogenous ET.

In addition to ERF10 (PGSC0003DMG400010285), which is involved in the plant hormone signaling transduction pathway, there were 12 ERFs including ERF1, ERF3, ERF5, and ERF-like genes (Table 4). Among these, ERF003-like gene PGSC0003DMG400014127, ERF3 gene PGSC0003DMG400022305, and ERF034-like gene PGSC0003DMG400026035 were down-regulated, and the other ten ERF coding genes were all up-regulated. ERF2-like gene PGSC0003DMG400002272 and ERF017 gene PGSC0003DMG400002899 had high fold-changes of 28.3 and 319, respectively, at 12 h of ET treatment compared with 0 h. In addition, PGSC0003DMG400011633 and PGSC0003DMG400016769 encoded StWRKY9 and STWRKY8, respectively, which were enriched in the plant-pathogen interaction pathway with up-regulated expression (Table 4).

**Table 4:** Differentially expressed genes that encoded ethylene-responsive factors in SD20.

Gene ID	Log_2_FC			Regulated			Annotation
	3 h	6 h	12 h	3 h	6 h	12 h	
PGSC0003DMG400002272	0	4.74	4.82	n	Up	Up	ERF2-like
PGSC0003DMG400002899	0	5.33	8.32	n	n	Up	ERF017
PGSC0003DMG400005837	2.16	1.23	0.94	Up	Up	n	ERF061-like
PGSC0003DMG400010285	0	0	3.27	n	n	Up	ERF10
PGSC0003DMG400010750	0.69	0.74	1.43	n	n	Up	ERF1-like
PGSC0003DMG400014127	− 2.36	− 0.26	-0.01	Down	n	n	ERF003-like
PGSC0003DMG400016003	1.98	2.49	3.68	n	Up	Up	ERF5-like
PGSC0003DMG400016006	1.93	0.96	1.15	n	n	Up	ERF1-like
PGSC0003DMG400022305	− 0.91	− 1.05	-0.61	n	Down	n	ERF3
PGSC0003DMG400026035	− 0.99	− 0.76	-2.24	n	n	Down	ERF034-like
PGSC0003DMG400026136	1.59	1.50	1.82	n	Up	n	ERF003-like
PGSC0003DMG400026232	0.32	2.36	4.87	n	Up	Up	ERF5-like
PGSC0003DMG400036566	− 0.58	0.60	3.05	n	n	Up	ERF5
PGSC0003DMG400011633	0.72	1.70	3.64	n	n	u	StWRKY9
PGSC0003DMG400016769	1.47	1.51	2.55	u	u	u	STWRKY8

### Differential expression of protein kinases response to ET

Out of the 1226 ET-specific DEGs, functional annotation showed that 82 genes encoded protein kinases (PKs) (Fig. 6A), which belonged to ten PK families. The largest group was serine/threonine protein kinase (STKs) with 32 members, followed by leucine-rich repeat receptor-like PKs (LRR-RLKs, 15 members), putative receptor protein kinases (PRPKs, four members), lectin-domain receptor protein kinases (three members), mitogen-activated protein kinases (MAPKs, two members), and calcium-dependent protein kinase (CDPK, one member) (Fig. 6B). Among 82 PK genes, 67% were up-regulated and two STK genes (PGSC0003DMG400004928 and PGSC0003DMG400025509) and one LRR-RLK gene (newGene_18308) were significantly up-regulated at 3 h; 37% of PK genes were not up-regulated until 6 h and 26% were not up-regulated until 12 h. The expression of LRR receptor-like serine/threonine-protein kinase FLS2 gene PGSC0003DMG400008296 continued to increase by 2–5 times at 3 h, 6 h, and 12 h of ET treatment. Two Avr9/Cf-9 induced kinase genes PGSC0003DMG400002327 and PGSC0003DMG400027893 were up-regulated and increased 7.8 and 8.7 times at 12 h, respectively.

### Differential expression of genes related to oxidoreductase activity and calcium ion signal transduction response to ET

In the GO enrichment analysis, a total of 92 DEGs were enriched in oxidoreductase activity (Fig. 6A), including oxygenase (34 members), oxidase (13 members), reductase (12 members), and dehydrogenase (nine members) (Fig. 6B). Out of 34 oxygenase genes, 23 genes (67.6%) encoded cytochrome P450 monooxygenase (CYP450), which contained CYP71, CYP76, CYP78, CYP88, CYP704, CYP736, CYP705, and CYP801 family members. Among 13 oxidase genes, five genes encoded ACO, the key enzyme in the ET synthesis pathway; four ACO genes were up-regulated.

A number of biosynthetic key enzyme DEGs were also identified that encoded gibberellin 20-oxidase 4 and ent-kaurenoic acid oxidase (KAO), the key enzymes in GA synthesis, and protein ECERIFERUM 3/WAX2 (CER3/WAX2), cinnamoyl-CoA reductase (CCR), and 3-dehydroquinate dehydratase/shikimate dehydrogenase isoform 2 (DHD/SHD). The last three enzymes are key enzymes in the biosynthesis of plant epidermal wax, lignin, and aromatic amino acids, respectively, and all three coding genes were up-regulated (Table 5). Pheophorbide a oxygenase (PaO) is the key enzyme in chlorophyll degradation and is encoded by the accelerated cell death 1 gene (ACD1) in *Arabidopsis*. In this study, PaO coding gene PGSC0003DMG400002967 was up-regulated after exogenous ET treatment. The key tropane alkaloids biosynthetic enzymes littorine mutase and alcohol dehydrogenase (ADH) genes were down-regulated (Table 5).

**Table 5:** Key enzyme genes involved in the synthesis of secondary metabolic substances.

Gene ID	Regulated	Annotation
PGSC0003DMG400000011	Up	Gibberellin 20-oxidase 4
PGSC0003DMG400001823	Up	Ent-kaurenoic acid oxidase, KAO
PGSC0003DMG400030998	Down	Alcohol dehydrogenase, ADH
PGSC0003DMG400025274	Down	Littorine mutase/monooxygenase
PGSC0003DMG400011170	Up	Protein ECERIFERUM 3/WAX2, CER3/WAX2
PGSC0003DMG400010000	Up	Cinnamoyl-CoA reductase, CCR
PGSC0003DMG400002967	Up	Pheophorbide a oxygenase, PaO
PGSC0003DMG400005284	Up	3-Dehydroquinate dehydratase/shikimate dehydrogenase isoform 2, DHD/SHD

In GO enrichment analysis, there were 19 DEGs in the enrichment of calcium ion binding, and 13 of them encoded calcium-binding protein, two genes encoded calmodulin, one gene encoded calcium-dependent protein kinase (CDPK), and the other three genes encoded thylakoid lumenal 29.8 kDa protein, annexin p35, and respiratory burst oxidase homolog protein C. Fourteen out of 19 DEGs were up-regulated.

### Differential expression of pathogenesis-related protein genes response to ET

A group of plant produced proteins induced by different stress stimuli, named “pathogenesis-related proteins” (PRs) are essential in plant defense against pathogens and in general adaptation to stressful environments. PR proteins include chitinase, glucanase, protease inhibitor, endoproteinase, and peroxidase in different plants. Out of 1226 DEGs, 17 genes encoded PRs (Fig. 6A), including five endo β-1,3-glucanase (PR-2), three chitinases (PR-3), three peroxidases (PR-9), two ribonucleases (PR-10), and one each of thaumatin (PR-5), proteinase inhibitor (PR-6), endoproteinase (PR-7), and defensin (PR-12) (Fig. 6B). The PR-6 coding gene newGene_12101 and peroxidase 65 gene PGSC0003DMG400020494 were down-regulated, and other PR genes were up-regulated.

### Verification of differential gene expression

**Figure 7:**
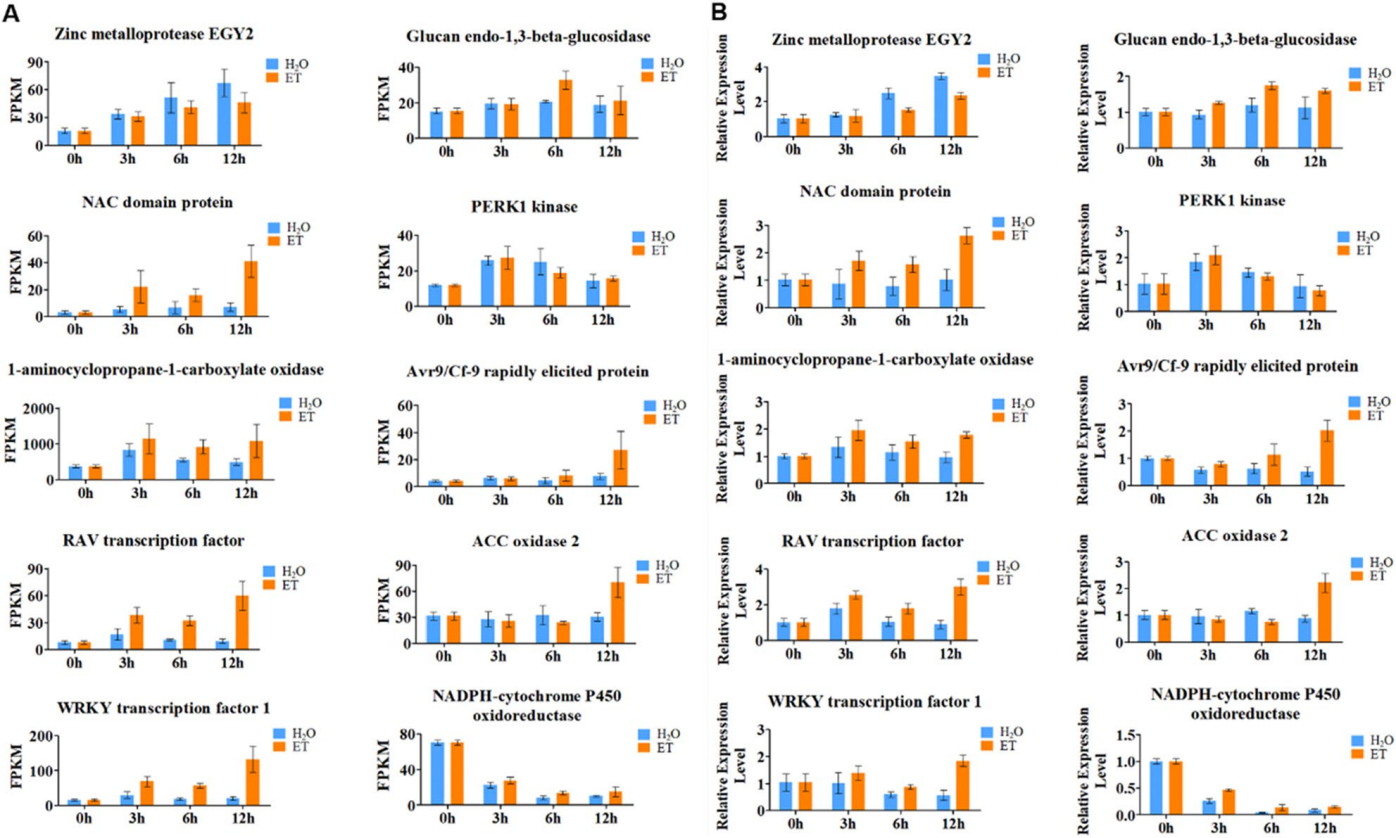
Gene expression profiles of RNA-seq (**A**) and experimental verification of gene expression levels by qRT-PCR (**B**).

In order to verify the accuracy of RNA-seq data, we randomly selected ten DEGs for qRT-PCR verification. The results showed that except for NADPH (down-regulated), the other nine genes were up-regulated: zinc metalloprotease EGY2, NAC domain protein, 1-aminocyclopropane-1-carboxylate oxidase, RAV, WRKY1, and PERK1 kinase coding genes were up-regulated at 3 h of ET treatment; glucan endo-1,3-beta-glucosidase gene was up-regulated at 6 h of ET treatment; while Avr9/Cf-9 rapidly elicited protein and ACC oxidase 2 genes were up-regulated at 12 h of ET treatment. This is consistent with the results of the transcripts determined by RNA-seq analysis (Fig. 7), indicating that our transcriptome analysis data is reliable.

### ET synthesis and defensive signal conduction in SD20 and key genes

**Figure 8:**
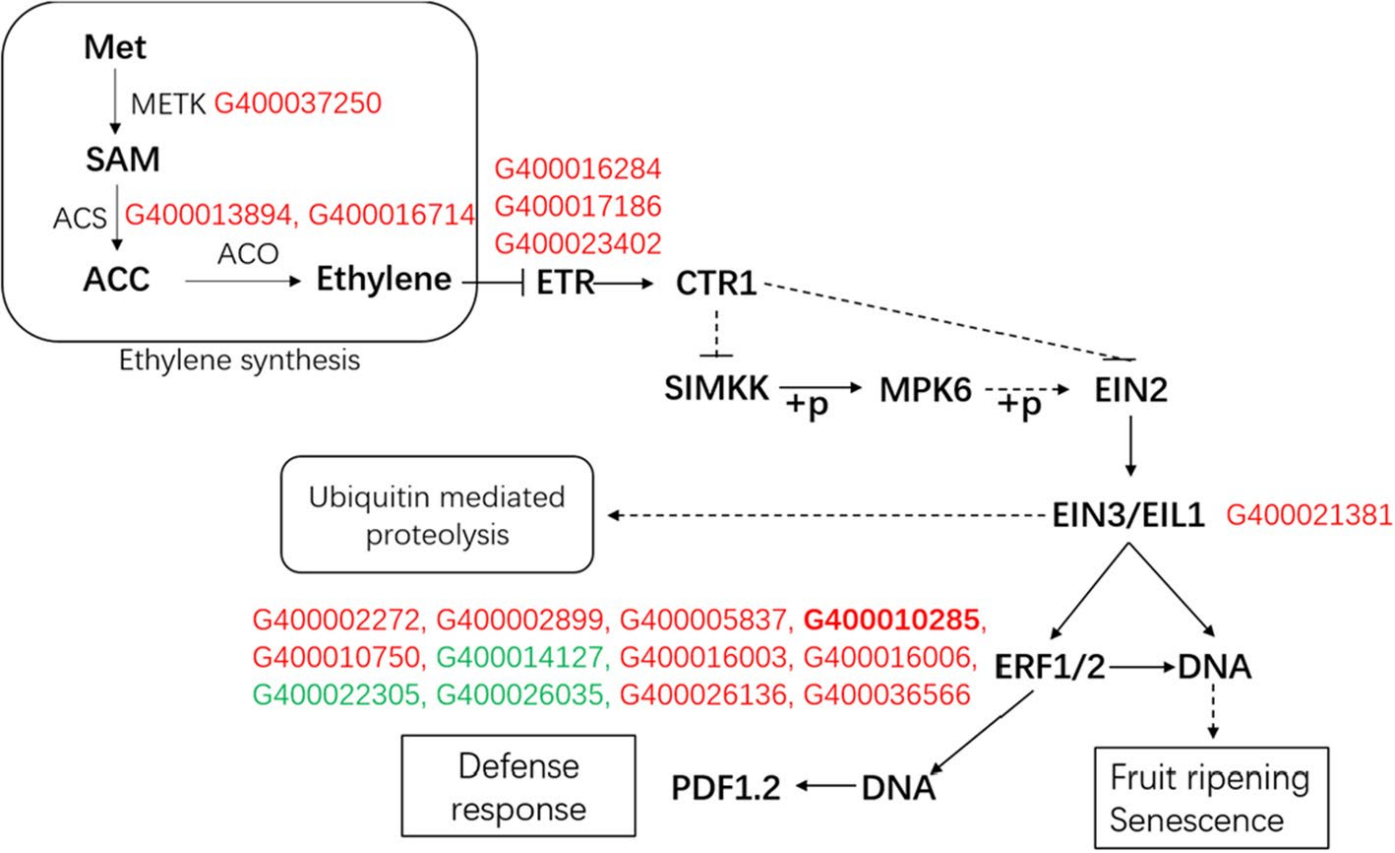
Pathways of ethylene synthesis and metabolism in potato genotype SD20 and key genes. Red in the figure represents the up-regulated genes, and green represents down-regulated genes. ‘G’ in the gene ID stands for ‘PGSC0003DMG’.

The key genes of the ET synthesis pathway include S-adenosylmethionine (SAM), ACC synthase (ACS) and ACC oxidase (ACO). The key genes in the ET signaling pathway include ETR, EIN2 (ethylene-insensitive2), EIN3 (ethylene-insensitive3), EIL (ethylene-insensitive3 like), and ERF1. In this study, one SAM synthetase gene METK2 (G400037250), two ACO genes (G400013894, G400016714), three ETR genes (G400016284, G400017186, G400023402), one EIN3 gene (G400021381), and 12 ERF genes were differentially regulated (Fig. 8). Except for three ERF genes (G400014127, G400022305, and G400026035) that were down-regulated, the other 16 genes had up-regulated expression. These key genes may be essential in the response to ET-induced signal transduction in SD20.

## Discussion

### Exogenous ET initiates the immune defense response pathway in SD20

In this study, 1226 genes were specifically differentially expressed under ET treatment in tetraploid potato SD20. The GO enrichment analysis showed that 1226 DEGs were enriched in 68 biological process terms, which were mainly related to the metabolism processes of lipid, indole derivative, and phosphate, and responses to exogenous stimulation, immunity, and defense. These results indicated that ET first initiated the response to stimulus, then mobilized the biological synthesis of various biological macromolecules, and finally regulated the expression of related genes through ion transport and signal transduction, which obviously induced the immune defense process, and may be related to disease resistance.

Further KEGG pathway analysis showed that SD20 was first stimulated by hormones, which initiated plant hormone signal transduction; second, hormone signal transduction initiated expression of immune defense-related genes, and enhanced the plant's defense capabilities; and finally, a series of metabolic processes that produced glutathione, nitrogen, sulfur, cysteine, and methionine were induced to maintain the normal growth and reproduction of SD20 plants. During this process, biosynthesis of secondary metabolites such as steroids, alkaloids, and plant toxins coordinated and promoted the adaptability of plants under stress stimuli. Secondary metabolites are not necessary for cellular life or plant growth and development, but the accumulation of secondary metabolites is related to plant resistance. In this study, the phosphatidylinositol signaling system (sot04070), which was critical in plant cell responses to environmental stress, was significantly enriched, indicating that ET can induce SD20 to improve resistance to stimulus and stress including pathogen infection.

### Exogenous ET stimulates differential expression of TFs, PKs, defense, and oxidoreductase-related genes involved in immunity and defense in SD20

In this study, genes of TFs, PKs, and a large number of immune defense genes were differentially expressed. It was speculated that exogenous ET was essential in enhancing resistance to stimulus or disease in SD20.

TFs are key factors in plant innate immunity. In this study, TFs DEGs mainly encoded zinc finger protein, ERF, and WRKY, including zinc finger protein Zat10, ERF1, ERF2, ERF5, WRKY6, WRKY11, and WRKY53. Many of them have been reported to function directly in plant defense^13^. The plant tolerance to drought, salt, and oxidative stress is improved by elevating the transcript level of Zat10 and regulating the expression of reactive oxygen removal genes^14,15^. ERF1 is co-activated by JA and ET signaling pathways to regulate the expression of downstream pathogen response genes^16^. ERF2 and ERF5 respond to different pathogens by coordinating several chitinase and other defensive pathways^17,18^. OsWRKY6 and OsWRKY53 are positive regulators for defense responses, and several defense-related genes, including PR genes such as OsPR10a and PBZ1, were activated in OsWRKY6- or OsWRKY53-overexpressed transgenic rice plants, finally resulting in enhanced resistance to *Magnaporthe grisea*^19,20^. Tobacco WRKY11 participated in plant PAMP-triggered immunity (PTI) and effector-triggered immunity (ETI) processes by interacting with NADPH oxidase RBOHB^21^. As above, the TFs found in this study should play a positive regulatory role in the response to exogenous ET treatment in SD20.

PKs can be used as pattern recognition receptors (PRRs) to identify specific molecules produced by pathogens or damaged plant tissues^22,23^, and are thus involved in signal transduction during defensive responses^24^. PKs found in this study included STKs, RLKs, and MAPKs. The LRR-RLK FLS2 is a widely available PRR in plants and initiates PTI through recognizing the conserved N-terminal 22-amino acid sequence (flg22) of bacterial flagellin^25,26^. FLS2 coding genes were up-regulated in our study indicating their positive regulatory role in SD20 response to ET.

Plant cells can produce numerous defense enzymes including glutathione S-transferases (GSTs) after they are stimulated by a variety of biotic and abiotic stresses^27^. GSTs protect plant cells from damage by detoxifying and antioxidant effects^28,29^. GST genes also respond to infection with fungi and other pathogens, and GSTU13 is an integral part of the immune pathway that produces the defensive indole glucosinolates. In *A. thaliana*, lack of functional *GSTU13* leads to increased susceptibility to several fungal pathogens^30^. In this study, the expression of seven GST genes was induced by ET, which is consistent with a previous study^31^, indicating that GSTs may play a protective role in improving resistance to external stimuli and antioxidants in SD20.

### Multiple signaling pathways participate in the response of SD20 to ET

The plant hormone signaling network is an important participant in the plant immune regulation network^32^, which combines antagonism and synergy to form a complex defense system^33^. The JA/ET pathway is primarily responsible for protecting against the infection of necrotrophic pathogens, while SA-mediated defense signaling pathways are activated after infection with biotrophic pathogens^34,35^. Crosstalk of these three hormones, along with other hormones such as ABA, IAA, BR, CTK, and melatonin, are also essential in plant disease prevention^36^. In this study, the top KEGG metabolic pathway was plant hormone signal transduction (sot04075) including IAA, CTK, ABA, ET, JA, and SA. The involved genes encoded hormone synthesis and signal transduction key genes including Aux/IAA, AP2/ERF TF, ethylene receptor, and EIN3/EILs. AP2/ERF TFs are the integration factors of the hormone signaling pathway and are essential in the SA-JA-ET signaling network^37^. EIN3/EILs are the nodes of ET, JA, and SA signaling pathways^38^. This indicated that ET not only activates the ERF1-induced defensive reaction in the ET signal pathway, but also activates the defense reaction of JA and SA signal-mediation in SD20.

The plant defensin 1.2 (PDF1.2) gene is specifically induced by JA or ET and the marker genes for the JA/ET dependent signaling pathway. In this study, one defensin-like protein 1 gene (PGSC0003DMG400032549) was significantly up-regulated. We also found that SA signal marker genes PR-2 (five DEGs), PR-5 (one DEG), and NPR1 (one DEG), JA signal pathway marker gene PR-3 (three DEGs), and JA synthesis key enzyme lipoxygenase (LOX) genes (two DEGs) were differentially expressed. Taken as a whole, ET, SA, and JA signal pathways are involved in the response to exogenous ET treatment and may be related to improved resistance in SD20. This is consistent with the results of multiple signals involved in disease resistance to pathogen infection in SD20^5^, and further supports the important role of ET in mediating resistance and defense in potato SD20. DEGs involved in IAA, GA, ABA, and other signal pathways have different signal transduction and regulation roles in the growth and development of SD20.

### Exogenous ET induces internal ethylene synthesis and defensive signal conduction in SD20

ET biosynthesis in plants begins with methionine (Met) and is first catalyzed by S-adenosylmethionine synthetase to produce SAM. Then, ET is formed by oxidation cracking of the important intermediate metabolite ACC. ACS and ACO are the key enzymes in ET biosynthesis. In our study, one SAM synthetase gene METK2 and two ACO genes were differentially up-regulated, which indicated that internal ET was synthesized and accumulated in SD20.

Normally the concentration of ET in the air is very low, and under these circumstances, ethylene receptor ETR1 (ETHYLENE RESPONSE 1) and CTR1 (CONSTITUTIVE TRIPLE RESPONSE 1) are combined together and then combined with phosphate downstream signal component EIN2, then EIN2 in the phosphorylation state is degraded by the ubiquinone/26S protease pathway, inhibiting the transmission of signals downstream. When ET concentration increases, ET molecules and receptors combine to inactivate CTR1 and cannot phosphorylate downstream EIN2, and the dephosphorylated ET insensitive EIN2 protein is freely transferred from the endoplasmic reticulum into the nucleus, promoting EIN3/EIN1 accumulation in the nucleus. EIN3/EIN1 can directly activate the DNA reaction or activate ERF1/2 to indirectly activate the DNA reaction to participate in the regulation of fruit maturation; on the other hand, EIN3/EIN1 directly activates ERF1/2, which can act on DNA to activate PR protein activity, and participate in defensive reactions^39^. In this study, three ETR genes and one EIN3 gene had up-regulated expression, which indicated an important positive regulatory role in response to ET-induced signal transduction in SD20 through directly or indirectly activating defensive response, hormone regulation, and ubiquitin–proteasome-mediated degradation.

### ET may also be involved in resistance and defense to biotrophic action of pathogens during early infection stages in SD20

SA signaling is generally considered to be involved in resistance to biotrophic pathogens, whereas ET/JA signaling is essential to stop infection by necrotrophic pathogens^40,41^. For example, ET treatment enhanced plant resistance to the necrotrophic fungus *Botrytis cinerea*^42^ and the necrotrophic bacterium *Erwinia carotovora*^43^. Other studies reported that *Arabidopsis EIN2* and *EIN3* mutants decreased the susceptibility to biotrophic pathogen *Heterodera schachtii* and hemibiotrophic pathogen *Pseudomonas syringae* pv. tomato^44,45^. Overexpression of tomato *ERF* transcription factors, Pti4, Pti5, and Pti6 enhanced the plant defense to *P. syringae* in tomato and biotrophic fungus *Erysiphe orontii* in *Arabidopsis*^46,47^. These results indicated that the effect of ET on plant disease resistance depends on the interactions of specific plants with pathogens.

*Phytophthora infestans* is a hemibiotrophic pathogen with biotrophic action during early infection and necrotrophic action in the later stages of colonization. A previous study reported that the susceptibility of young *Nicotiana benthamiana* plants to *P. infestans* is due to a lack of induction of SA signaling, while the resistance of mature plants against this pathogen requires both SA-regulated appropriate induction of cell death and ET-induced production of phytoalexin^48^. We compared 1226 DEGs induced by ET with the DEGs that were specific to *P. infestans* race CN152 at 24 h, 48 h and 72 h in potato SD20^5^. The results showed that the DEGs with high fold-change under pathogen infection were also differentially expressed under exogenous ET treatment and had the exact same expression trends, which included the up-regulated PR-2 gene PGSC0003DMG400029830 and fatty acid desaturase gene PGSC0003DMG400036004 at 24 h; up-regulated p-coumaroyl quinate/shikimate 3′-hydroxylase gene PGSC0003DMG400007179 and SlTCP3 gene PGSC0003DMG400015377; and down-regulated cytochrome P450 gene PGSC0003DMG400026523 at 72 h. Also, two LOX genes (PGSC0003DMG400010859 and PGSC0003DMG400024693), and one ACO gene (PGSC0003DMG400013894) were up-regulated at 24 h after CN152 infection in SD20. This indicated that ET signaling may also be involved in resistance to the biotrophic action of *P. infestans* during early infection stages in SD20.

## Conclusion

Here, we performed transcriptome analysis of exogenous ET-treated potato leaves in the high late blight resistant potato genotype SD20 using RNA-seq technology, and the main objectives were to reveal the role of exogenous ET in the defense pathway and the specific responses of SD20 to ET. We found ET stimulated the defense response and initiated a similar defense pathway compared to pathogen infection in SD20. Multiple signaling pathways including ET, SA, and JA were involved in the response to exogenous ET in the host SD20. Our results lay a solid foundation for further understanding of the ET signaling transduction pathway and its mechanisms in disease defense systems in potato.

## Materials and methods

### Plant materials

Tetraploid potato cultivar SD20 with high resistance to potato late blight was used in this study. Tissue cultured plants of SD20 were grown in glass bottles (72 × 59 mm) containing 30 mL MS medium supplemented with vitamins and 30 g·L^−1^ sucrose. Plants were cultivated at 22 °C under a 16 h light/8 h dark cycle, with eight seedlings per bottle.

### Exogenous ethylene treatment

Three-week-old aboveground seedlings of SD20 were sprayed with 0.2 mmol·L^−1^ ethephon (C_2_H_6_ClO_3_P, Cat^#^E8021, Solarbio), an ethylene releasing compound or with an equal amount of sterile water as a control. Each treatment was repeated three times and aboveground seedlings were harvested at 0 h, 3 h, 6 h, and 12 h. A total of 21 samples were frozen in liquid nitrogen immediately for RNA extraction and analysis.

### Library construction and RNA sequencing

These methods were based on the published articles with some modifications^11,49^. Total RNA was extracted using the RNAprep Pure Plant Kit (Tiangen Biotech, Beijing, China) according to the protocol provided and treated with DNase I to generate 21 RNA libraries. RNA integrity was assessed with the 2100 BioAnalyzer instrument using the Agilent RNA 6000 Nano Kit (Agilent Technologies, Santa Clara, CA, USA). One μg of RNA was used to generate a sequencing library with the NEBNext Ultra RNA Library Prep Kit (NEB, USA) according to the manufacturer's recommendations, and the library quality was checked on an Agilent 2100 BioAnalyzer system. Libraries were sequenced using the HiSeq X Ten system (Illumina Inc., USA) with the 150-cycle paired-end sequencing protocol.

### Analysis of RNA-Seq datasets

Raw data were filtered and reads containing adapters, poly-N sequence, or low-quality reads were removed to obtain clean reads. All downstream analyses were based on high quality clean reads and Q20, Q30, and GC content of clean data were calculated. These clean reads were then aligned to the potato reference genome sequence using HISAT2 software^50^. The mapped reads were assembled using StringTie^51^. Alternative splicing (AS) types and their corresponding expression quantities were generated by ASprofile software^52^. FPKM (Fragments Per Kilobase of transcript per Million fragments mapped) was used as an indicator for measuring the level of transcript or gene expression^52^. The reference accession, the doubled haploid *S. tuberosum* Group *Phureja* clone DM1-3 516R44 (hereafter referred to as DM) genome sequence (SolTub 3.0) and annotation files were downloaded from the ENSEMBL plants database (ftp://ftp.ensemblgenomes.org/pub/plants/release-34/fasta/solanum_tuberosum/dna/)^53^. Raw data in FASTQ format are available in the Genome Sequence Archive (GSA) in the BIG DATA Center, Beijing Institute of Genomics (BIG), Chinese Academy of Sciences, under the accession number CRA002320.

### Identification of differentially expressed genes

DESeq was used to normalize expression levels of genes and perform differential expression analysis based on the negative binomial distribution^54^. Genes with normalized expression fold-change greater than 2 and false discovery rate (FDR) less than 0.05 were considered to be differentially expressed. Differentially expressed genes (DEGs) were annotated based on the functional annotation information of ENSEMBL release *Solanum tuberosum* SolTub_3.0 and the potato ortholog *Arabidopsis* genes.

### GO and KEGG enrichment analysis of differentially expressed genes

Gene Ontology (GO) enrichment analysis of DEGs was performed using agriGO (http://bioinfo.cau.edu.cn/agriGO/analysis.php) based on a hypergeometric test. KOBAS software (http://kobas.cbi.pku.edu.cn/kobas3) was used to test the statistical enrichment of DEGs in KEGG (Kyoto Encyclopedia of Genes and Genomes) pathways^5,55^. GO and KEGG terms with corrected *p* values < 0.05 were considered to be significantly enriched.

### Validation of RNA-Seq data by real-time quantitative PCR (qRT-PCR)

To validate the results of the RNA-seq data, ten DEGs were selected randomly for qRT-PCR. The RNA samples were identical to the samples used for RNA-seq analysis. Two μg of total RNA was used per 20 μL reaction for reverse transcription. The PCR system was 20 μL containing 10 μL of SYBR Premix *Ex* Taq (Takara, Japan), 0.5 μL of forward and reverse primers, 7 μL of double-distilled water, and 2 μL of the cDNA gene. All qRT-PCR reactions were performed with an annealing temperature of 60 °C and a total of 40 amplification cycles with three replicates for each cDNA sample. The expression level of each gene was calculated using the 2^−ΔΔCt^ method with GADPH as an internal reference gene^56^. Primers (Supplementary Table S1) were designed using PRIMER 5^57^.

## Supplementary information

Supplementary Information.

## Data Availability

The datasets generated and/or analyzed during the current study are available from the corresponding author on reasonable request. Raw RNA-seq data from the study have been deposited in the Genome Sequence Archive (GSA) under accession number CRA002320.
