# Supplementary material for: Transcriptome analysis reveals that exogenous ethylene activates immune and defense responses in a high late blight resistant potato genotype
Source: Sci Rep. 2020 Dec 4;10:21294. doi: 10.1038/s41598-020-78027-5 (PMC7718909; doi:10.1038/s41598-020-78027-5)
Supplement: Supplementary file 1 — Supplementary Information. [file 41598_2020_78027_MOESM1_ESM.docx]

Transcriptome analysis reveals that exogenous ethylene activates immune and defense responses in a high late blight resistant potato genotype

Xiaohui Yang^1,†^, Li Chen^1,2,†^, Yu Yang^1^, Xiao Guo^1^, Guangxia Chen^1^, Xingyao Xiong^2^, Daofeng Dong^1*^ & Guangcun Li ^2*^

^1^ Institute of Vegetables and Flowers, Shandong Academy of Agricultural Sciences/Molecular Biology Key Laboratory of Shandong Facility Vegetable/National Vegetable Improvement Center Shandong Sub-Center/Huang-Huai-Hai Region Scientiﬁc Observation and Experimental Station of Vegetables, Ministry of Agriculture and Rural Affairs, Jinan 250100, China, ^2^ Institute of Vegetables and Flowers, Chinese Academy of Agricultural Sciences/Key Laboratory of Biology and Genetic Improvement of Tuber and Root Crop, Ministry of Agriculture and Rural Affairs, Beijing 100081, China

| Gene | Forward Primer Sequence (5’-3’) | Reverse Primer Sequence (5’-3’) | Gene Description |
| --- | --- | --- | --- |
| PGSC0003DMG400028078 | ACATTCGGTCTTGTCCTG | CGGAGAAACCCGTAAGTC | zinc metalloprotease EGY2 |
| PGSC0003DMG400032555 | CGAATCATCGGAGTCAAT | TTCTGTGGTCTTCGTCCC | NAC domain protein |
| PGSC0003DMG400021476 | GCAGTTTCCACAGCCTTC | TGGAGCACAGGGTGATTG | 1-aminocyclopropane-1-carboxylate oxidase |
| PGSC0003DMG400000811 | GGGTTTGGTTAGGCACAT | GGTATCACAAGCCTATTAAGTT | RAV transcription factor |
| PGSC0003DMG400028520 | CCTCAGGACCAACCATTA | GTCCAGCAAGATCCCATT | WRKY transcription factor 1 |
| PGSC0003DMG400000354 | TGGAGGAGTTGGCGTTAG | GGATTACATTGGGTAGGTTG | Glucan endo-1,3-beta-glucosidase |
| PGSC0003DMG400000379 | AGTTTGGTGGACTGGGTG | CGTGGTCTACGCTTTGC | PERK1 kinase |
| PGSC0003DMG400030364 | AACCCTTACCCTAACTCACT | CAGAATATCCATCACCCTCA | Avr9/Cf-9 rapidly elicited protein |
| PGSC0003DMG400016714 | CAACTATCCTCCTTGTCCC | GCTATTGACATCCTAGTGCC | ACC oxidase 2 |
| PGSC0003DMG400013779 | TCGGGGTATTCAACGC | CAAGGAGGGAAGGGAGA | NADPH-cytochrome P450 oxidoreductase |
| GAPDH | CTGGTGCTGACTTCGTCG | CTGGCTTGTATTCATTCTCG | Internal reference gene |

**Supplementary Table S1.** Primer sequences used for quantitative real-time PCR.
